# The diagnostic performance of non-contrast T1-mapping in patients with acute myocarditis on cardiovascular magnetic resonance imaging

**DOI:** 10.1186/1532-429X-14-S1-P179

**Published:** 2012-02-01

**Authors:** Vanessa Ferreira, Stefan K Piechnik, Erica Dall'Armellina, Theodoros Karamitsos, Jane M Francis, Robin P Choudhury, Attila Kardos, Matthias G Friedrich, Matthew D Robson, Stefan Neubauer

**Affiliations:** 1 Cardiovascular Medicine, University of Oxford, Oxford, UK; 2 Stephenson Cardiovascular MR Centre, Libin Cardiovascular Institute of Alberta, University of Calgary, Calgary, AB, Canada; 3 Cardiology, Milton Keynes NHS Hospital Foundation Trust, Milton Keynes, UK; 4 Cardiology, Université de Montréal, Montréal, QC, Canada

## Summary

Non-contrast T1-mapping using ShMOLLI can serve as a novel CMR diagnostic criterion in patients presenting with suspected acute myocarditis.

## Background

The accurate diagnosis of acute myocarditis on cardiovascular magnetic resonance imaging (CMR) often requires multiple modalities, including T2-weighted (T2w), early and late gadolinium imaging. T1-mapping is an emerging technique which is also sensitive to acute changes in free water content. We hypothesized that non-contrast T1-mapping using the novel Shortened Modified Look-Locker Inversion Recovery (ShMOLLI) sequence can serve as a new diagnostic criterion for acute myocarditis.

## Methods

**Figure 1:**
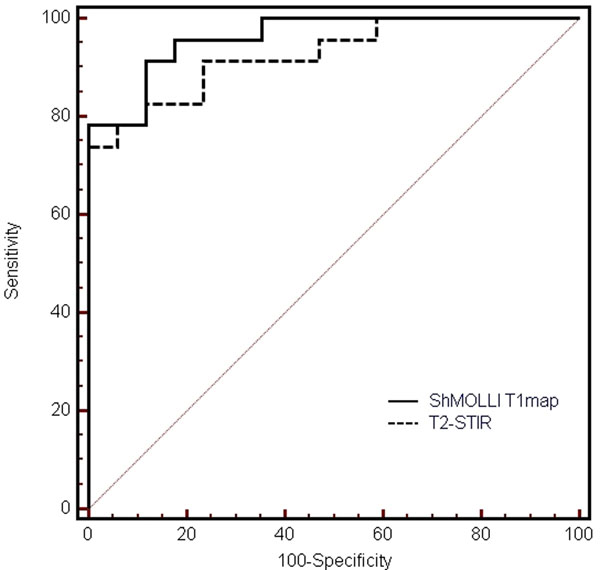
ROC curves for ShMOLLI T1-mapping and T2-STIR in acute myocarditis.

We studied 23 patients with suspected acute myocarditis and 17 healthy controls. All patients presented with chest pain and troponin I > 0.04 ug/L. CMR within 7 days included (1) T2w short-TI inversion recovery (STIR);(2) ShMOLLI T1-mapping; and (3) phase-sensitive late gadolinium enhancement (LGE) (Fig 1). T2 signal intensity (SI) relative to skeletal muscle (T2 SI ratio) and absolute T1 values per-subject were analyzed.

## Results

**Figure 2:**
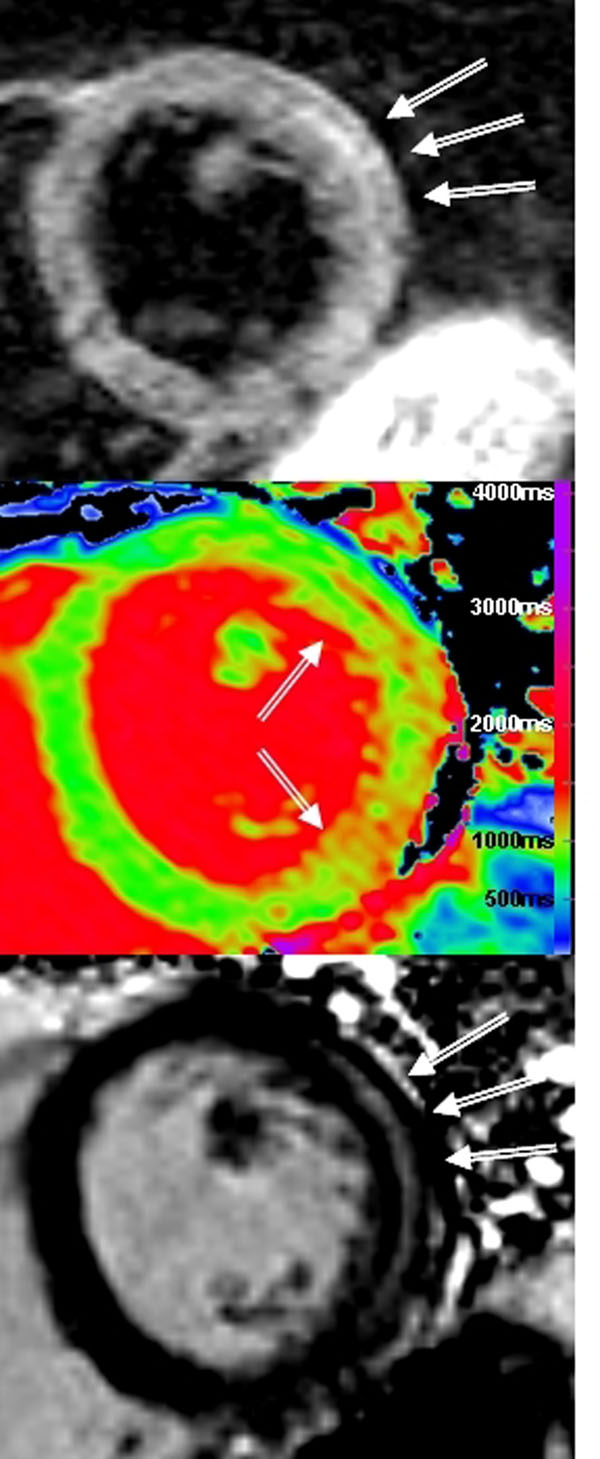
Acute myocarditis. (Top) STIR demonstrating increased signal intensity in the mid lateral wall. (Middle) ShMOLLI T1-map demonstrating increased T1 values (1100-1200 ms) in the lateral wall. (Bottom) LGE imaging demonstrating mid-wall enhancement in the lateral wall.

All patients had a CMR diagnosis of acute myocarditis based on both positive T2-STIR and typical LGE pattern. Compared to controls, both mean T1 and T2 SI ratio in patients were significantly higher (T1=1036±71ms vs. T1=938±19; T2 SI ratio=1.77±0.24 vs. 1.52±0.10, p<0.0002 for both). Receiver operator characteristics analysis showed excellent diagnostic performance for both methods: the area-under-the-curve for ShMOLLI T1-mapping=0.96 and STIR=0.93 (p=0.3, Fig 2). The equal sensitivity and specificity points were T1=87% (T1=958ms) and T2=83% (T2 SI ratio=1.63).

## Conclusions

Non-contrast T1-mapping using ShMOLLI has a high diagnostic performance for acute myocarditis and may be used as a novel additional CMR diagnostic criterion.

## Funding

This study is funded by the Oxford National Institute for Health Research Biomedical Research Centre Programme. VMF is funded by the Alberta Heritage Foundation for Medical Research (AHFMR) and the University of Oxford Clarendon Fund Scholarship. Dr. Robin Choudhury is a Wellcome Trust Senior Research Fellow in Clinical Science. Stefan Neubauer and Robin Choudhury acknowledge support from the British Heart Foundation Centre of Research Excellence, Oxford.

